# Development of Elective Flexibility and Related Cognitive Functions Across Three Cultures

**DOI:** 10.1111/desc.70299

**Published:** 2026-11-03

**Authors:** Sarah Pope‐Caldwell, Elisa Felsche, Ardain Dzabatou, Wilson Vieira, Luke Maurits, Dominik Deffner, Daniel Haun

**Affiliations:** ^1^ Department of Comparative Cultural Psychology Max Planck Institute for Evolutionary Anthropology Leipzig Germany; ^2^ Department of Psychology Georgia State University, Atlanta, Georgia; ^3^ Language Research Center Georgia State University, Atlanta, Georgia; ^4^ Department of Arts and Humanities Université Marien Ngouabi Brazzaville Republic of the Congo; ^5^ Department of Psychology Marburg University Marburg Germany; ^6^ Center for Adaptive Rationality Max Planck Institute for Human Development Berlin Germany; ^7^ Department of Human Behavior Ecology and Culture, Max Planck Institute for Evolutionary Anthropology Leipzig Germany; ^8^ Science of Intelligence Excellence Cluster Technical University Berlin Berlin Germany

**Keywords:** cognitive flexibility, decision‐making, elective flexibility, executive functioning, lifespan cognitive development

## Abstract

Elective flexibility, the ability to switch between working strategies adaptively, is critical for decision‐making in complex environments. Here we experimentally assessed elective flexibility alongside three related cognitive measures short‐term memory, inhibition, and responsive flexibility–in 325 children and adults (ages 4–81‐years) from three cultures. Elective flexibility performance improved with age for Germans and, to some extent, for Congolese BaYaka and Bandongo participants. Under stable conditions, younger German children were more explorative than older children and adolescents. Next, using interviews with 62 adults from these three cultural groups we found evidence that cultural variation in daily exposure to unpredictable variability corresponded with more elective switching whereas exposure to harshness corresponded with less. By combining richly contextualized cross‐cultural and developmental data with multiple cognitive tests and computational models, this research uniquely contributes to our understanding of the development of children's flexible decision‐making skillsets.

## Summary

- Here we examined the lifespan developmental trajectories of elective flexibility, responsive flexibility, short‐term memory, and inhibition across three cultures in Germany and the Republic of the Congo.
- We found age‐related improvements in elective flexibility, as well as striking cross‐cultural differences in the use of reinforcement learning approaches on a four‐armed bandit decision‐making task.
- We contextualized these findings using interviews, noting that cultural variation in exposure to variability, predictability, and harshness corresponded with variation in elective flexibility.

## 1 Introduction

Adaptive decision‐making requires a difficult, if not impossible, balance between adopting useful strategies and exploring, or even switching to, alternatives. In a perfect world, where all strategies and their outcomes are known, one might simply choose the best option. However, in most real decision‐spaces, we are confronted with many possible strategies, often with unknown outcomes, that require time and energy to sample. Successfully navigating these decisions requires cognitive flexibility—the ability to adaptively select between known solutions and novel alternatives in a contextually appropriate manner (Laureiro‐Martínez and Brusoni 2018; Pope 2018; Ueltzhöffer et al. 2015).

Although cognitive flexibility is considered a core facet of executive functioning—alongside working memory, and inhibition (Brydges et al. 2014; Diamond 2013; Friedman and Miyake 2017)—it has most often been studied under “forced‐switch” conditions (Pope‐Caldwell 2024). In forced switch paradigms, a previously effective strategy stops working, thereby necessitating a change in behavior (e.g., Bunge and Wallis 2008; Doebel and Zelazo 2015). For example, in the Wisconsin Card Sorting Task (WCST), a hallmark measure of cognitive flexibility, participants must infer based on feedback (correct vs. incorrect) that a sorting rule has changed (Berg 1948). The need to switch strategies within forced‐switch paradigms may arise endogenously, meaning that it is internally identified—such as within the WCST—or exogenously, meaning that participants are explicitly told that the rule has changed—such as in the Dimensional Change Card Sort Task (Forstmann et al. 2005; Frick and Chevalier 2023; Snyder and Munakata 2010; Zelazo 2006; see also Jacques and Zelazo 2013). We have argued, however, for a broader distinction between *responsive flexibility* and *elective flexibility* (Pope‐Caldwell 2024). Responsive flexibility refers to shifting away from a previous strategy that no longer works, regardless of whether that need is externally cued or internally identified (e.g., both WCST and DCCS). Elective flexibility refers to choosing whether to shift away from a still‐functional—but potentially suboptimal—strategy. For example, in Luchins’ (1942) famous water‐jar task, participants were instructed to add and subtract the amounts of water contained in three hypothetical jars in order to reach a target quantity. Participants learned to use a tedious 4‐step strategy [e.g., Jar 2 (42 units)—Jar 1 (9 units)—Jar 3 (6 units)—Jar 3 (6 units) = target quantity (21 units)] but then were given trials in which a two‐step shortcut was also available [e.g., long solution: Jar 2 (49 units)—Jar 1 (23 units)—Jar 3 (3 units)—Jar 3 (3 units) = target quantity (20 units); shortcut: Jar 1 (23 units)—Jar 3 (3 units)]. Luchins found that remarkably few participants “electively” switched to the more efficient, but novel, shortcut.

Both responsive and elective flexibility draw on overlapping cognitive resources—including short‐term memory, to track strategy values; updating, to revise those values based on experience; and inhibition, to suppress suboptimal responses. However, elective flexibility places substantially greater demands on these processes. Because multiple strategies remain viable simultaneously, elective decisions require maintaining and comparing concurrent options, evaluating opportunity costs, and tolerating uncertainty—a computational burden absent from responsive flexibility tasks, in which the decision space is reduced to a single working strategy at a time.

Developmentally, research on cognitive flexibility has largely focused on responsive flexibility paradigms. Between ages 3 and 5, children show substantial improvements in exogenous responsive flexibility—the ability to switch strategies when cued (Blakey et al. 2016; Frick et al. 2021; Smidts et al. 2004; Zelazo 2006; Zelazo et al. 1996). Performance on responsive flexibility tasks is also positively associated with working memory and inhibition, even in childhood (Blakey et al. 2016; Friedman and Miyake 2017; Miyake et al. 2000; Mullane and Corkum 2007; Nweze and Nwani 2020). Thus, responsive flexibility develops early and is tightly linked to other related cognitive functions (Diamond 2013).

Far less is known about the development of elective flexibility. Although elective switching behavior has been studied across fields—including decision science, psychology, and behavioral ecology—these literatures are rarely considered in tandem, and few studies have assessed elective flexibility explicitly across development. What evidence exists points in conflicting directions, likely reflecting the heterogeneity of paradigms, populations, and outcome measures studied—as well as the inherently dynamic contexts in which elective flexibility is employed. Infancy and childhood are often characterized as periods of heightened information gathering and strategy sampling (Bonawitz et al. 2012; Bridgers et al. 2019; Bruner 1972; Cusack et al. 2024; Gopnik 2020; Gweon and Schulz 2008; Legare 2012; Lew‐Levy and Amir 2026; Thompson‐Schill et al. 2009). Across a range of paradigms, children exhibit more elective flexibility—indicated by a greater willingness to explore alternative solutions—than adults (German and Defeyter 2000; Gopnik et al. 2015; Liquin et al. 2021; Luchins 1942; Mata et al. 2013). For example, Liquin and Gopnik (2022) found that in a “learning trap” task, where exploiting an obvious answer leads to a wrong conclusion, preschoolers and early school‐aged children explored more than adults and learned the true structure of the environment better. Similarly, Pope et al. (2015) found that children ages 7–10‐years‐old used a novel shortcut more than adolescents (11–18 years) or adults (19–68 years), who preferred a familiar but inefficient strategy. Yet other findings point toward developmental increases in elective flexibility. Cunningham (1965) reported improvements in children's ability to identify and adopt more efficient strategies between ages 7 and 11. Similarly, Hopper et al. (2020) found greater flexibility in 4‐year‐olds relative to 2‐year‐olds on a problem‐solving task. Age‐related increases in flexible strategy‐use are also apparent in domains such as tool innovation (Cutting et al. 2014; Lew‐Levy et al. 2021; Neldner et al. 2019) and insight problem‐solving (Cheke et al. 2012). Taken together, developmental trajectories of cognitive flexibility are neither uniformly increasing nor decreasing. Rather, they appear to depend on the specific form of flexibility measured and the contextual structure in which it is embedded.

The theoretical basis for high levels of elective flexibility and exploration in childhood is that it allows children to sample many options, serving the eventual goal of homing in on the best ones. Yet this is necessarily context dependent. In Pope‐Caldwell (2024)’s Constrained Flexibility Framework, elective flexibility is posited to be (i) reduced under harsh, stable or predictably variable conditions, but (ii) enhanced under unpredictable conditions, as long as the risk of failure is low. From a developmental perspective, we might expect that, in a stable environment, where strategy efficacy does not change substantially over time, high exploration in early childhood might be replaced by exploitation over the course of development (Pope‐Caldwell 2024). Indeed, this is consistent with empirical findings from search tasks where rewards are distributed in a stable, predictable manner (Giron et al. 2023). However, in more variable environments, where strategy efficacy might change frequently, a continued willingness to explore, even into adulthood, should remain adaptive. In the Constrained Flexibility Framework, harshness exerts an overarching suppressive effect on elective flexibility. Indeed, Pope‐Caldwell et al. (2024) found that experimentally induced “harshness” reduced elective exploration in English‐speaking adults. Similarly, Vieira et al. (2025) observed that elective shortcut‐use increased with age in German participants, decreased with age among Congolese BaYaka participants, and remained stable among Congolese Bandongo participants—which they suggest may correspond with varying degrees of market integration, where higher integration serves as a buffer to the risks of trying unfamiliar strategies (Amir et al. 2020). Indeed, Vieira et al. (in press) found that German children were more explorative than Congolese children in a small‐scale foraging task, and that risk tolerance increased as rewards accumulated for both German and Congolese children, although the number of rewards that were needed before this buffering effect was observed was higher for Congolese children. Thus, understanding how cognitive flexibility develops, especially elective flexibility, requires understanding how variation in the contexts in which children are acquiring their decision‐making skills shapes their later deployment.

## 2 The Current Study

In this study, we asked: (i) How does elective flexibility develop, and how does it relate to responsive flexibility, short‐term memory, and inhibition? and (ii) How is elective flexibility shaped by ecological and cultural context? Interview and experimental data were collected with participants (ages 4–81) across three cultures: BaYaka and Bandongo living in the Republic of the Congo and participants residing in Germany. The three communities sampled here represent only a small subset of the diversity of human experience; however, they differ substantially along important cultural dimensions—such as subsistence style, formal schooling, and market integration—which shape the stability, variability, and harshness of their daily problem‐solving demands.

To answer question one, participants completed a computerized task battery, consisting of (1) an elective flexibility game (**Bandit Jars**), (2) a responsive flexibility (set‐switching) game (Color‐Shape Shifting Task; **CSST**; *not done in Congo), (3) an inhibition game (Early Childhood Inhibitory Touchscreen Task; **ECITT**), and (4) a visuospatial short‐term memory game (Corsi Block‐Tapping Task; **Corsi**). See Figure 1 and full descriptions below. These games were chosen through iterative discussions with Congolese and German collaborators and after several phases of piloting (more details below).

**FIGURE 1:**
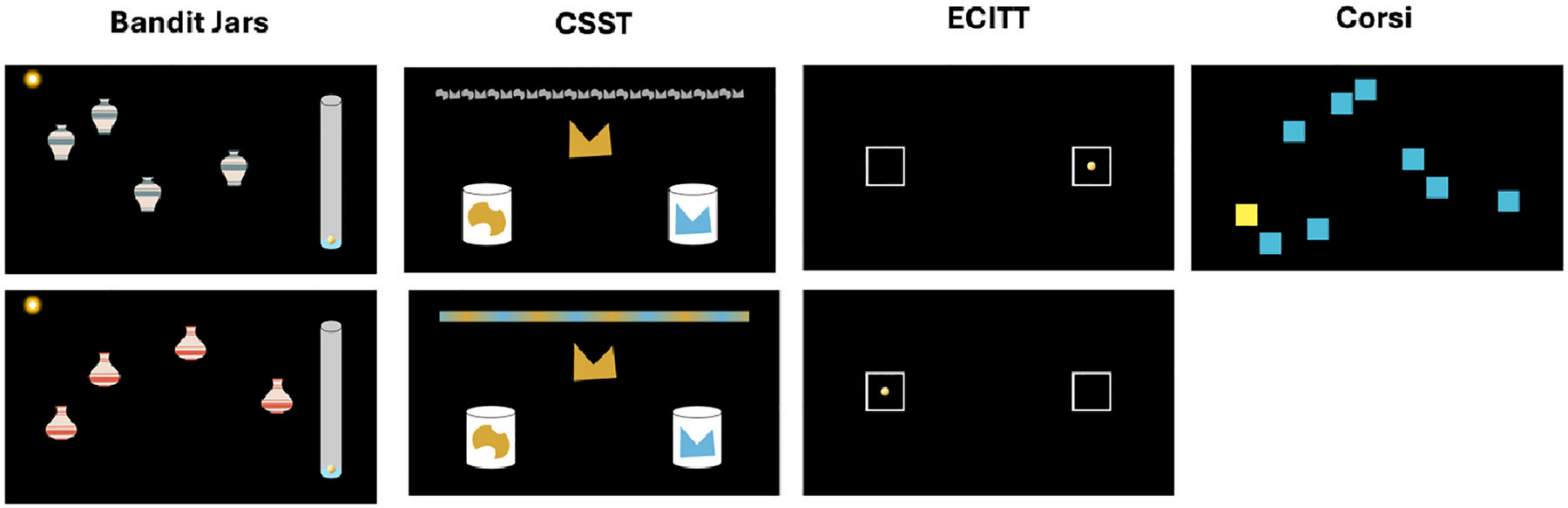
Example images from the Bandit Jars Task, Color‐Shape Shifting Task, Early Childhood Inhibitory Touchscreen Task and the Corsi Block‐Tapping Task.

To better contextualize any cross‐cultural variation in our experimentally derived measures of elective flexibility and related cognitive functions, we used broad indicators of community‐level exposures to variability, predictability, and harshness based on free‐response interviews with a subset of adult participants. Questions aimed to characterize community‐level aspects of predictability, variability, and harshness in participants’ daily activities and food acquisition (e.g., “What kind of food will you buy/collect today?”, “What did you eat today?”, and “Did you have any problems or feel like you were in danger today?”). We acknowledge that this method does not capture the breadth of, nor individual variation in, all participants’ daily problem spaces; however, it allowed us make broad inferences without over‐burdening our participant pool.

### 2.1 Ethnographic Context

In the Republic of the Congo, the study was conducted in a multi‐ethnic village in the Likouala Department. The village is a day's travel from the nearest town and only accessible by boat. At the time of data collection, there were ∼420 Bandongo fisher‐farmers and ∼480 BaYaka foragers living in the village. There is no cellular service in the village and limited exposure to computers or touch‐screen devices outside of ongoing research, including the present study.

Bandongo primarily subsist on horticultural gardening of small plots in the rainforest surrounding the village, fishing on the river and in seasonally flooded areas of the forest, and hunting using traps and guns (Kandza et al. 2023). Bandongo engage in monetary exchanges for market goods at small shops in the village and a biweekly market boat Lew‐Levy et al. 2025). Bandongo social structure is patriarchal and gender and age stratified (Boyette et al. 2018). Bandongo children have much autonomy over their daily activities, and frequently engage in autonomous play and learning in multi‐aged groups—although completion of assigned household tasks is expected by caregivers. There is a primary school in the village and Bandongo children are encouraged by their parents to engage with formal education (Xu et al. 2026), although access to additional schooling requires that children leave the village. Indeed, in a 2019 survery only ∼50% of Bandongo adults in the village reported completing primary school (Pope‐Caldwell 2019).

BaYaka primarily subsist by foraging fruit, tubers, greens, mushrooms, seeds, insects, and nuts, fishing in seasonally flooded areas of the forest, and hunting using traps, spears, and guns (Benoit et al. 2024; Kitanishi 1995; Veen et al. 2023). BaYaka infrequently engage in the local market economy; however, they sometimes work for Bandongo in exchange for money or goods Lew‐Levy et al. 2025). BaYaka are egalitarian, with little gender or age stratification and sharing of food and other resources is both highly valued and expected (Gettler et al. 2023; Lew‐Levy et al. 2026). BaYaka children engage in formal education infrequently, due in part to linguistic and financial barriers; in a 2019 survey almost no BaYaka adults in the village reported completing primary school (Pope‐Caldwell 2019). BaYaka children have a great deal of autonomy over daily activities, and frequently engage in autonomous play and learning in multi‐aged groups, with few required or forbidden activities.

In Germany, data collection occurred in an urban center with a population of ∼600,000 in the state of Saxony. The city is home to Germans, Germans with migration backgrounds (∼6%), and foreigners (∼13%) (City of Leipzig, Office for Statistics and Elections 2023); although this study did not collect data on primary citizenship, all participants spoke fluent German. Only ∼3% of residents were unemployed at the time of data collection, corresponding to strong market integration. Indeed, food is primarily obtained via purchase at grocery stores and restaurants, and other resources are purchased from shops, markets, and online. Compulsory schooling begins at age 6.

### 2.2 Study Aims and Hypotheses

We preregistered the following hypotheses (https://osf.io/vmbkz). First (H1), under variable conditions, we predicted a U‐shaped pattern of elective switching across age, with young children (4–8) and adults (18+) switching more frequently than older children (9–12) and adolescents (13–17). This prediction is grounded in evidence that early childhood is characterized by heightened random exploration and broad strategy sampling (Gopnik 2020; Ionescu 2017; Pope et al. 2015), whereas adulthood permits more strategically deployed exploration calibrated to environmental demands (Giron et al. 2023). In contrast, late childhood and adolescence may represent a transitional period marked by reduced exploration (Giron et al. 2023), but not yet fully mature, context‐sensitive flexibility. Under stable conditions, we predicted a negative association between age and switching, reflecting both decreasing exploratory tendencies across development and the reduced adaptive value of exploration in stable environments. Second (H2), we predicted that elective flexibility would become more skillful with age—that is, characterized by increasingly effective switching—and would be positively associated with responsive flexibility, short‐term memory, and inhibition in childhood (4–12), with weaker associations in adolescence and adulthood. Third, consistent with the hypothesis that elective flexibility is tuned to daily decision‐making environments, we predicted that (H3.1) greater exposure to environmental variability would be associated with more elective switching, whereas (H3.2) greater exposure to environmental harshness would be associated with reduced switching, regardless of variability. Finally, we expected elective switching to be best predicted by an interaction between age, culture, and condition, such that young children would show similar patterns across cultures, with culturally specific norms emerging later—by late childhood in stable conditions and adolescence in variable conditions.

## 3 Method

### 3.1 General Information

Testing took place in Fall 2021‐Spring 2022 in Leipzig, Germany and the Likouala region of the Republic of the Congo, with German and Congolese BaYaka and Bandongo participants. All experimental procedures were approved by the Ethics Council of the Ethics Council of the Max Planck Society (Appl. No: 2021_45). Tasks were chosen and developed to be accessible to all participants; for example, tasks did not require literacy, numeracy, or cultural knowledge specific to any one community. That said, by using gamified experimental tasks we did not avoid known cross‐cultural variation in participants’ experience with abstract, rule‐guided games nor their comfort‐level with one‐on‐one, adult‐guided testing scenarios, both of which German participants were far more familiar with. Initial piloting of the experimental games was done virtually with adult research assistants in Congo, and with children in Germany—due to the logistical barriers of conducting a full piloting season in the Congolese village (e.g., financial, seasonality of research seasons, and the already limited number of potential participants). Upon arrival in the village, we further piloted the games with Congolese children and adjusted translations and procedures accordingly. All consent, assent, interview and experimental scripts were translated into participants’ primary language: Yaka for BaYaka, Lingala for Bandongo, and German for Germans. Translations and back‐translations were done separately, by collaborators and members of the research team fluent in the local dialects for each language, with discrepancies discussed and final wording agreed upon by both translators and co‐authors SPC and AD.

Congolese participants were recruited by SPC, co‐authors EF, AD, and WV, and other members of our team of local Congolese research assistants. Upon arrival in the village, a meeting was held to explain the study and research goals in detail. Additionally, the consent procedure was explained–clearly stating that prior to any data collection, we would give each child and adult the opportunity to decide not to participate and that this decision could be made at any point during data collection. After receiving permission to conduct the research from the village leaders, we went from household to household, re‐explaining the research agenda and answering any questions. Both interested, and non‐interested households received a large cooking pot at the end of the field season, regardless of participation, and this was explicitly stated both during the general meeting and in household meetings. For interested participants and families, we obtained consent with a verbal script—translated into Yaka for Bayaka participants and Lingala for Bandongo participants and witnessed by two research team members. Both parental/guardian consent and child assent were obtained for all participants that were younger than 18 and unmarried. Informed consent was obtained for all participants that were older than 18 or married. Testing occurred at the dedicated research station in the village. Interviews relating to a subset of adult participants' daily activity and subsistence variability, predictability, and harshness were conducted in a convenient location of participants' choosing, often near participants’ households.

German participants were recruited through the Max Planck Institute for Evolutionary Anthropology's Comparative Cultural Psychology department's child research lab. Participants already registered in the child research lab database were contacted via telephone and, after a brief study explanation, were asked if they, or their child, would be interested in participating. Testing took place at the Max Planck Institute for Evolutionary Anthropology, after parental consent and child assent were obtained. In accordance with normal testing procedures, at the end of testing, children received a small toy and adults received a chocolate bar. Interviews relating to a subset of adult participants' daily activity and subsistence variability, predictability, and harshness were conducted over the phone.

### 3.2 Experimental Games

#### 3.2.1 Participants

For the experimental games, data were collected from 92 Bandongo, 69 BaYaka, and 170 German participants. Data were not analyzed for participants who did not complete the Bandit Jars Task [1 Bandongo (8 years‐old); 3 BaYaka (15, 32, and 6 years‐old); 1 German (5 years‐old)] or whose data were lost due to technical malfunction (1 German (19 years‐old)). It was not possible to collect CSST data in Congo (see supplementary discussion). Our final dataset consisted of 91 Bandongo [N_female_ = 42, ages 4.0–71.0 years, mean (SD) = 19.7 (14.6)], 66 BaYaka [N_female_ = 29, ages 3.5–62.1 years, mean (SD) = 19.4 (14.5)], and 168 Germans [N_female_ = 85, ages 4.0–81.4 years, mean (SD) = 18.4 (14.7). See Figure S1 for details. Congolese data collection was terminated once all interested participants had the opportunity to participate up until the end of the 2‐month research season. German data collection was terminated once the planned number of participants had been tested. For all tasks, participants were required to pass an initial training and comprehension phase, with instructions repeated up to three times before training was considered unsuccessful and the session was discretely ended.

#### 3.2.2 Computerized Games

***Bandit Jars: A measure of elective flexibility***. Participants tried to find the most water by choosing between four identical water jar graphics, randomly positioned, but not overlapping, on the screen. The amount of water dispensed by each jar ranged from 0 to 100 units and either (1) changed substantially over time (*variable* condition) or (2) stayed roughly the same over time (*stable* condition). Participants completed two 50 trial blocks, one in each condition; presentation order was counterbalanced across participants. For each trial, participants could either continue to use the same jar they used in the previous trial, or they could switch to any of the other three jars. We generated five sets of stable and variable reward structures (see Pope‐Caldwell et al. 2024; and https://github.com/sarahpopecaldwell/HarshFlex_BanditJars), presentation of which was counterbalanced across participants. Collected water was automatically added to the “tube” on the right side of the screen. Each time the tube was filled, a physical bead was dispensed into the participants’ cup using a customized 3D‐printed dispenser (https://github.com/ccp‐eva/bead‐dispenser#overview) based on Maurer et al. (2016).

***CSST: A measure of responsive flexibility***. Participants were taught to sort a target stimulus into one of two buckets based on color, then shape, then by switching between these two rules by following a cue–a banner across the top of the screen–indicating the active rule (Friedman et al. 2016; Nweze and Nwani 2020). Each successful sort yielded one real bead.

***ECITT***: ***A measure of inhibition***. Participants first learned to touch the screen to “collect” an image of a bead that appeared. Then they completed 32 trials in which the bead appeared on one (the *prepotent*) side in 75% of trials, and on the other (the *inhibitory*) side in 25% of trials (Holmboe et al. 2021). The prepotent side was counterbalanced across participants. For each collected bead in the game, a physical one was dispensed.

***Corsi: A measure of short‐term memory***. Participants tried to remember the order in which a series of squares changed color. Squares were randomly positioned, although not overlapping, on the screen. The number of squares participants needed to remember increased over time until they failed twice within a level, indicating that their maximum memory capacity had been reached, up to nine squares (Diamond 2013).

See https://osf.io/xj7av/files/osfstorage for all protocol, analyses scripts, and experimental game files. Note, all beads were traded for a small prize at the end of the testing session.

#### 3.2.3 Interviews

We conducted interviews twice a day, in the morning and evening, on three non‐consecutive days with 62 adults: 23 BaYaka (N_female_ = 11, Age_mean_ = 35.7^1^, Age_range_ = 15.0–67.1 years), 19 Bandongo (N_female_ = 11, Age_mean_ = 37.8, Age_range_ = 19.7–64.0 years), and 20 Germans (N_female_ = 12, Age_mean_ = 34.3, Age_range_ = 18.6–53.4 years). Roughly half of these interviewees also participated in the experimental task battery (6 BaYaka, 8 Bandongo, and 13 Germans). Ideally, we would have collected interviews from all participants; however, we opted to recruit a representative subset of adults from each community to reduce the already immense time burden on participating families.

Morning interviews were scheduled so as to occur before any eating or general activities [Mean(sd) Interview 1 time: BaYaka – 6:39 a.m. (15 min); Bandongo – 6:50 a.m. (24 min); Germans – 8:28 a.m. (41 min)]. Evening interviews were scheduled so as to occur after most eating or general activities [Mean(sd) Interview 2 time: BaYaka – 6:30 p.m. (15 min); Bandongo – 6:42 p.m. (17 min); Germans – 7:18 p.m. (47 min)]. In the Republic of the Congo, interviews were done in‐person, often near participants’ homes, and in conjunction with GPS tracking (see Supporting Information). In Germany, interviews were conducted over the phone. Morning questions consisted of: (1) “What kind of activities will you do today?” and (2) “What kind of food will you buy/collect today?” Evening questions consisted of: (3) “What kind of activities did you do today?”, (4) “What kind of food did you buy/collect today?”, (5) “What did you eat today?”, (6) “Did you have any problems or feel like you were in danger today?” and (7) “Did you and your family eat enough today?”

In Congo, participant responses were translated during the interview by a local research assistant and co‐author AD and summarized in English by co‐author WV. In Germany, interview responses were summarized in German by child research lab research assistants (see acknowledgements: KS, LP, SS, MS, and PG) who then later translated them into English. All responses were first screened for relevancy and simplified into single items/actions by author SPC. For example, in response to the question “What kind of food will you eat today?”, a participant's response was originally recorded as “not planned to go out for eating, salad for lunch, dinner: carrot soup and baguette.” This was simplified to “salad, carrot soup, baguette.” See Supporting Information for an overview of all items/actions by categories: vegetable, meat, grain, drink, multipart dish, sweet, and so forth.

### 3.3 Analyses

#### 3.3.1 Development of Elective Flexibility

We assessed the developmental trajectory of elective flexibility and how it is shaped by culture, using a series of Bayesian GLMM regressions modelled using the *brms* package (Bürkner 2017) in R and weakly informative priors (see the OSF repository for all analyses scripts), predicting four measures of performance on the Bandit Jars task: (i) switching (Models 1.1‐3 in the OSF repository), (ii) score (Models 2.1‐3), (iii) elective exploration (Models 3.1‐2), and (iv) learning rate (Models 4.1‐2), for each condition (Stable, Variable). For age contrasts, we subset participants into four age groups, Young Children (∼3–8), Older Children (∼9–12), Adolescents (∼13–17), and Adults (∼18–81). Whenever possible, we used date‐of‐birth (or month/year if the full date was unavailable); however, for many Congolese participants date‐of‐birth was unknown. We then relied on age estimates based on community birth order (see Diekmann et al. 2017 for details) when available or consensus estimates with guidance from local research assistants otherwise.

We originally planned to run models which included the ECITT, Corsi, and (for German participants only) CSST scores; however, due to their collinearity with age (Figure S4), we deviated from the pre‐registered model formulae (see Supplementary Methods). Models 1–4.1 served as baseline models and included the fixed effect of sex and the random intercept of subject ID. Models 1–4.2 added fixed main effects of child age (modeled as a spline function, separately by condition), adult age, and condition, along with random slopes for condition by subject. Models 1–4.3 were run for analyses including all three cultural groups, these models further allowed the child age spline to vary by the interaction of condition and culture, with additional random slopes for age group and condition by culture to account for cultural variability in developmental trajectories. We determined the best‐fitting models by comparing their widely applicable information criterion (WAIC) values (McElreath 2020; Watanabe 2010).

#### 3.3.2 Switching and Performance Scores

Bandit Jars switching was calculated as the number of times participants switched from selecting one jar to another. Switching values could range from 0 to 49 switches per condition (Stable and Variable), and a binomial response distribution was used accordingly. Bandit Jars switching values indicate participants’ general propensity to change strategies, with higher values indicating more switching, regardless of whether switching was—or was not—beneficial.

Bandit Jars performance scores were calculated as the cumulative amount of water collected in each condition. Recall that with each jar selection, participants gain between 1 and 100 water units. This amount was converted to a proportion between 0 and 1 and a beta response distribution was used. In the stable condition, higher Bandit Jars scores indicate a greater ability to find and maintain a working strategy, whereas in the variable condition, higher scores indicate a greater ability to find and use higher yielding alternatives within a changing reward structure.

#### 3.3.3 Elective Exploration, Learning Rate, and Response Strategy

During data collection, it became clear that participants completed the Bandit Jars task using different underlying approaches. As anticipated, some participants used a value‐based, reinforcement learning (RL) approach (i.e., choosing jars using experience‐based latent values that are updated over time). However, some participants used alternative approaches, such as a “repeat” strategy, repeating the same jar selection throughout the trial block or a “random” strategy, where selections were seemingly chosen at random or following spatial patterns (e.g., selecting each jar in a circular motion and continuing that pattern throughout the task).

To better understand participants’ underlying response strategies on the Bandit Jars task, we ran a Bayesian multi‐level mixture model, which simultaneously inferred the (mixture) probabilities that participants in each community had used RL (θ_RL_), Repeat (θ_Repeat_), or Random (θ_Random_) candidate strategies, while also inferring subject‐specific RL parameters, that is, measures of elective exploration λ_k,l,j_ and learning rate φk,l,j, for each condition *k* (Stable and Variable; for previous implementations of Bayesian RL models, see (Deffner et al. 2020; Pope‐Caldwell et al. 2024). As these mixture probabilities all lie between 0 and 1, a beta response distribution was once again used for all GLMMs examining variation in response strategies across condition, age, and culture.

The elective exploration parameter characterizes the degree to which participants chose jars that aligned with learned jar preferences (i.e., were expected to yield high value rewards, based on their recent experiences with each jars’ payoffs). Lower elective exploration values correspond to more explorative selections. The learning rate parameter represents the speed at which participants update their preferences for each jar based on their recent experiences with each jars’ payoffs. Higher learning rates correspond to faster learning.

Given that elective exploration and learning rate parameters assume that participants are using an RL strategy, we planned to report analyses from (i) all participants (θ RL ≥ 0), (ii) those who were likely to have used an RL strategy (θ RL ≥ 0.5), and (iii) those who almost certainly used an RL (θ RL ≥ 0.9). However, in setting θ RL to greater than or equal to 0.5, our participant pool was reduced to: 0 BaYaka, 5 Bandongo, and 158 Germans; and θ RL ≥ 0.9 further reduced it to: 0 BaYaka, 0 Bandongo, and 89 Germans. Thus, we were unable to confidently analyze the elective exploration and learning parameters for Congolese participants, as planned (see discussion for possible explanations regarding cross‐cultural differences in Bandit Jars approaches). We instead focused on German participants’ exploration and learning rate values; however, setting Theta RL to ≥ 0.9 also limited our ability to assess developmental changes; as it left only two Young Children and three Older Children in the Variable condition. Thus, deviating from our pre‐registered analysis plan, we decided to use θ RL ≥ 0.8 to designate participants that we were confident (rather than nearly certain) were using an RL approach: 147 German participants, including 10 Younger Children and 9 Older Children in the variable condition.

#### 3.3.4 Response Strategy and Culture

To better understand how the likelihood of participants’ using a reinforcement learning strategy (*θ RL > 0.8)* differed over the course of development and across cultural group and condition, we ran an unplanned series of Bayesian logistic regressions to predict RL‐use. We chose *θ RL > 0.8* as the cutoff based on both the structure of the underlying data, which exhibited two distinct peaks (Figure S2a), as well as our choice (made prior to these analyses) to designate participants with theta RL values above 0.8 as confidently RL‐users. Models 5.1‐3 predictors were identical to Models 1–4; however, model priors were updated to account for the binomial structure (see data analysis scripts for details).

#### 3.3.5 Development of Elective Flexibility, Responsive Flexibility, Inhibition, and Short‐Term Memory

To investigate how elective flexibility develops in relation to other cognitive functions, we ran two exploratory analyses. First, we ran multivariate changepoint models to estimate the age at which each outcome variable experiences the greatest developmental change, for Bandongo (Model 6), BaYaka (Model 7), and German (Model 8) participants. The changepoint age was constrained using an inverse logit transformation to fall within a plausible range: 5‐ to 30‐years‐old. Second, we ran Kendall's rank correlation (*r*_τ_) analyses to examine associations between Bandit Jars performance and cognitive functioning measures within each age group and across cultures (Figure S5). Correlations stemming from groups with fewer than 10 participants (BaYaka younger children: *N* = 6, BaYaka older children: *N* = 7; Bandongo younger children: *N* = 9) were not interpreted, nor correlations between conditions or outcomes within the Bandit Jars task (although we include all analyses and results).

#### 3.3.6 Predictability, Variability, and Harshness Exposure

**Predictability**. As a proxy for predictability in participants’ subsistence and daily activities, we compared participants’ morning interviews to their evening interviews to identify when a planned food item or activity was, and was not, accomplished. Note, we used simplified labels for each item to ensure that all matches were identified, even if the exact phrasing differed between morning and evening interviews. We ran a series of multinomial regressions to predict the likelihood of an item being categorized as (1) “Planned and obtained/accomplished,” (2) “Obtained / Accomplished but not planned,” or (3) “Planned but not obtained / accomplished.” Models 9–10.1 included sex as a predictor, with subject and date as random effects. Models 9–10.2 also included culture as a predictor.

**Variability**. As a proxy for variability in participants’ subsistence and daily activities, we calculated whether each obtained food item or accomplished activity had already been obtained or accomplished by that participant, regardless of day. In this way, each food item and activity was coded as 1 (unique) or 0 (repeated). For example, if the participant reported going to fish twice one day and again on another day that they participated in the interviews, only the first instance would be coded as 1 (unique), the second and third would be both coded as 0 (repeated). We ran a series of logistic regressions to predict the likelihood that each obtained food or accomplished activity was unique (1) or repeated (0). Models 11–12.1 included sex as a predictor, with subject and date as random effects. Models 11–12.2 also included culture as a predictor.

**Harshness**. As a proxy for exposure to harshness in participants’ subsistence and daily activities, we coded whether participants reported that either they or their families had enough to eat (food harshness = 0) or if neither they nor their families had enough to eat (food harshness = 1). We also coded whether participants reported experiencing any problems or conflicts during their day (activities harshness = 1) or not (activities harshness = 0). Models 14–15.1 included sex as a predictor, with subject and date as random effects. Models 14–15.2 also included culture as a predictor.

## 4 Results

First, we examine the development of elective flexibility on the Bandit Jars task, including how frequently participants switched jars, how well they scored, and their underlying strategies as determined by their reinforcement learning model parameters (exploration, learning rate, RL‐use) across age and culture. We then compare developmental timelines across measures of elective flexibility (Bandit Jars score), responsive flexibility (CSST score), short‐term memory (Corsi score), and inhibition (ECITT score) via changepoint analyses, followed by exploratory within‐age correlations between Bandit Jars performance and the other tasks. Finally, we characterize cross‐cultural differences in food/activity predictability, variability, and harshness to contextualize these developmental patterns.

### 4.1 Development of Elective Flexibility

#### 4.1.1 Bandit Switching

Participants’ number of jar switches during the Bandit Jars task were best predicted by Model 1.2, which did not include the interaction between condition and culture. In the Stable condition, when each jar's value remained relatively stable throughout the trial block, we found that, across cultures, young children switched jars significantly more often than adolescents and adults [13.37 (1.90, 23.87)_Adolescents;_ 14.28 (2.87, 24.73)_Adults_]. Similarly, older children switched jars significantly more often than adolescents [7.17 (0.54, 14.37)_Adolescents_]. There were no significant age or cultural differences in switching in the Variable condition. See Figure 2.

**FIGURE 2:**
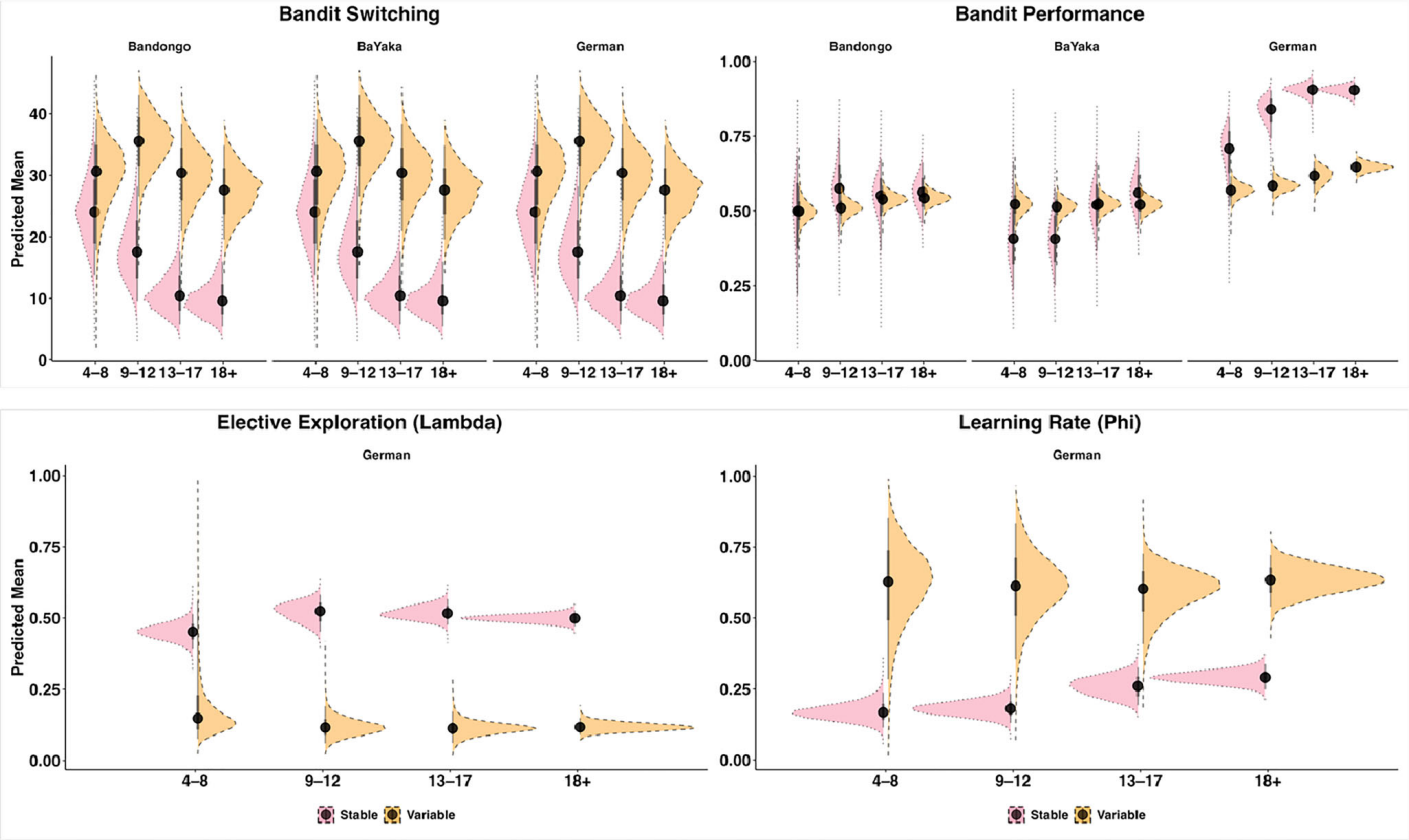
Posterior estimates plots of Bandit Jars Task results displaying participants’ (a) likelihood of switching jars, (b) overall score, (c) elective flexibility (lambda), and (d) learning rate (phi) across age groups and cultures—when applicable.

#### 4.1.2 Bandit Jars Performance

Participants’ Bandit Jars performance scores were best predicted by Model 2.3, which included the interaction between condition and culture. In the Stable condition, we found that German older children, adolescents, and adults outperformed younger children [Posterior predictive mean (95% CI) = 0.14 (0.04, 0.24)_Older Children;_ 0.21 (0.11, 0.31)_Adolescents;_ 0.21 (0.11, 0.31)_Adults_]. Similarly, adolescents and adults outperformed older children [0.07 (0.02, 0.13)_Adolescents;_ 0.07 (0.01, 0.13)_Adults_]. There were no other significant age differences in the Stable condition. In the Variable condition, we found that German adolescents and adults outperformed younger children [0.05 (0.00, 0.09)_Adolescents_; 0.08 (0.04, 0.12)_Adults_] and German adults outperformed older children and adolescents [0.06 (0.02, 0.11)_Older Children_; 0.03 (0.00, 0.06)_Adults_]. There were no other significant age differences in Bandit Jars scores in the Variable condition. Thus, in both Stable and Variable conditions, younger German children achieved lower scores than their older counterparts. We saw similar, but nonsignificant, improvements with age in Bandongo and BaYaka participants’ Stable condition performance, although these should be cautiously interpreted. In both Stable and Variable conditions, and across age groups except for young BaYaka and German children, German participants had higher Bandit Jars scores than BaYaka and Bandongo participants. See Figure 2.

#### 4.1.3 Elective Exploration

As noted before, we were unable to confidently analyze Congolese participants’ elective exploration and learning rates (although we include all analyses and results https://osf.io/xj7av/files/osfstorage). Here we report results from German participants who we were confident were using a reinforcement learning strategy, defined as *θ* RL > 0.8. Elective exploration (λ_k,l,j_) was best predicted by Model 3.2, which included all main effects and found that in the Stable condition, younger children were more explorative than older children [Posterior predictive mean (95% CI) = 0.07 (0.02, 0.12)] and adolescents [0.07 (0.02, 0.12)]. There were no significant age differences in the Variable condition. See Figure 2. Thus, we saw a U‐shaped trajectory for German participants’ elective exploration in the Stable condition.

#### 4.1.4 Learning Rate

Learning rate was also best predicted by the main effects model, Model 4.2. We found that in the Stable condition, German adolescents’ and adults’ learning rates were faster than both younger [Posterior predictive mean (95% CI) = 0.09 (0.03, 0.15)_Adolescents_; 0.12 (0.05, 0.19)_Adults_] and older [0.08 (0.03, 0.13)_Adolescents_; 0.11 (0.05, 0.17)_Adults_] children. There were no significant age differences in the Variable condition. See Figure 2. Thus, in line with their Bandit Jar scores, we observed a roughly linear increase in German participants’ learning rates with age, in the Stable condition.

#### 4.1.5 Reinforcement Learning Strategy

The probability of using a reinforcement learning strategy was best predicted by Model 5.3, which included the interaction between condition and culture. In the Stable condition, adolescent and adult German participants used a reinforcement learning strategy more than younger [Posterior predictive mean (95% CI) = 0.42 (0.26, 0.58)_Adolescent_; 0.43 (0.27, 0.58)_Adult_] and older [0.11 (0.02, 0.21)_Adolescent_; 0.11 (0.01 0.23)_Adult_] children, and older children used a reinforcement learning strategy more than younger children [0.31 (0.14, 0.48). In the Variable condition, again, adult and adolescent German participants used a reinforcement learning strategy more than younger [0.28; (0.09, 0.44)_Adolescent_; 0.38 (0.19, 0.56)_Adult_] and older [0.20 (0.04, 0.35)_Adolescent_; 0.31 (0.11, 0.50)_Adult_] children. There were no significant age differences for Bandongo and BaYaka participants, in either condition.

Across cultural groups, German participants, for all age groups, were more likely to use a reinforcement learning strategy than Bandongo participants, in both Stable [0.95 (0.86, 0.99)_Adults_; 0.94 (0.87, 0.96)_Adolescents;_ 0.84 (0.73, 0.93)_Older Children;_ 0.52 (0.36, 0.68)_Young Children_] and Variable [0.61 (0.46, 0.74)_Adults;_ 0.50 (0.38, 0.62)_Adolescents;_ 0.30 (0.18, 0.44)_Older Children;_ 0.22 (0.10, 0.37)_Young Children_] conditions, as well as BaYaka participants in both Stable [0.94 (0.83, 0.99)_Adults;_ 0.94 (0.86, 0.98)_Adolescents;_ 0.84 (0.72, 0.93)_Older Children;_ 0.52 (0.36, 0.68)_Young Children_] and Variable [0.61 (0.45, 0.74)_Adults;_ 0.50 (0.38, 0.62)_Adolescents;_ 0.30 (0.18, 0.44)_Older Children;_ 0.23 (0.11, 0.37)_Young Children_] conditions. See Figure S2b.

### 4.2 Development of Elective Flexibility, Responsive Flexibility, Inhibition, and Memory

#### 4.2.1 Developmental Changepoints Across Cultures

Exploratory analyses of the estimated developmental changepoints for elective flexibility (Bandit Jars performance in stable and variable condition), short‐term memory (Corsi), responsive flexibility (CSST), and inhibition (ECITT), indicated that, for German participants in the Stable condition, Bandit Jars performance reached an adult‐like plateau somewhat later than ECITT performance [Posterior predictive mean = −4.80 years (−0.54, −9.07)]. Germans’ ECITT scores also reached a developmental change point earlier than Corsi [−8.45 years (−3.55, −13.30)] and CSST [−4.54 years (−0.07, −8.87)] scores. No other contrasts were significant, although it should be noted that Germans’ Bandit Stable trajectory looked extremely similar to that of their CSST scores [0.26 years (−4.22, 4.72)]. See Figure 3.

**FIGURE 3:**
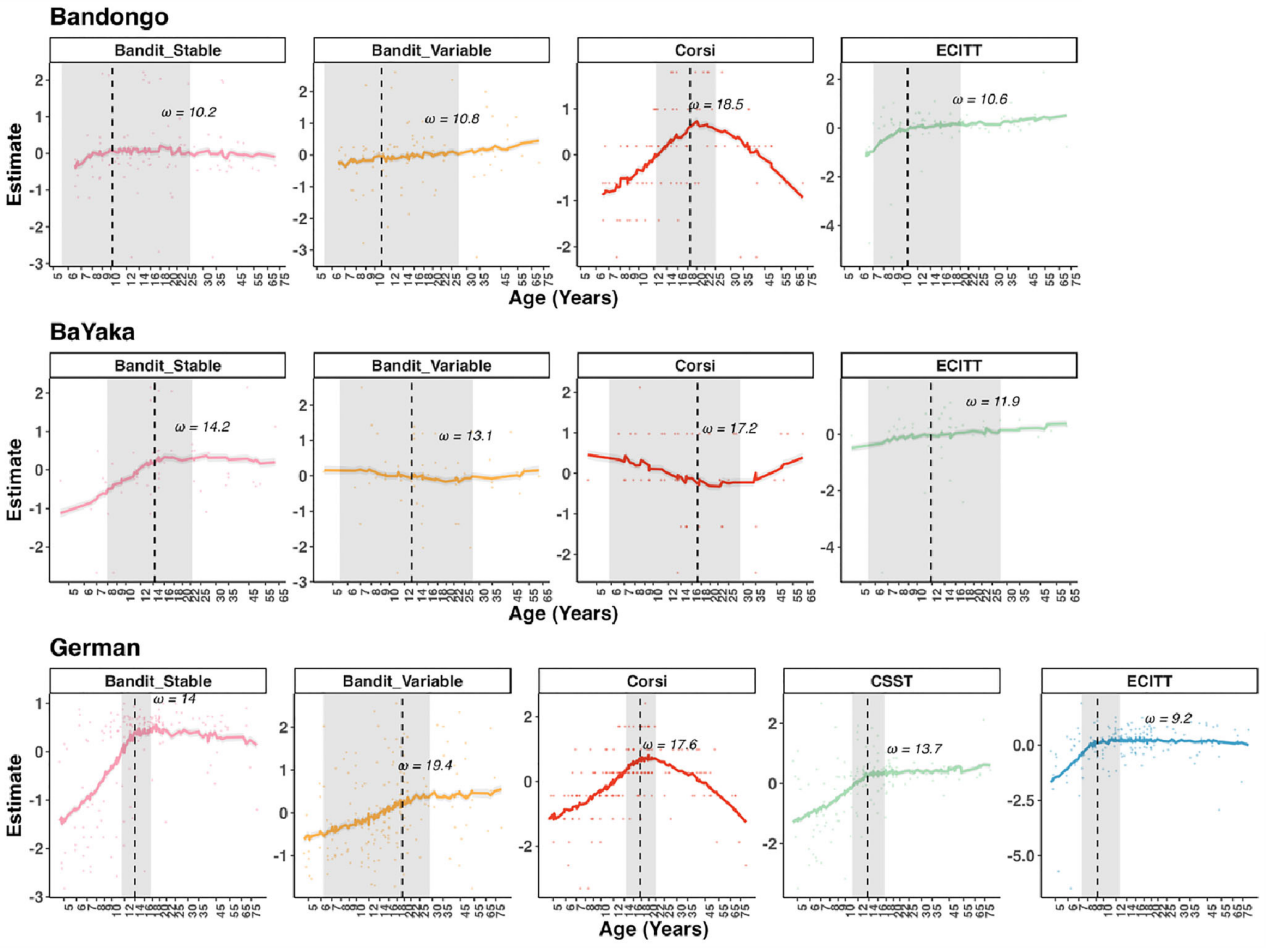
Changepoint analyses for the development of elective flexibility (Bandit Stable and Bandit Variable), memory (Corsi), responsive flexibility (CSST), and inhibition (ECITT), across cultures. Shaded areas represent 95% credible intervals. Note, CSST data were only collected with German participants.

#### 4.2.2 Correlations Across Cultures Within Age Groups

Exploratory correlation analyses indicated a slight negative correlation between young German children's switching (CSST) and Bandit Jars scores in the stable condition (*r_τ_* = −0.30, *p* = 0.03; Figure S5), suggesting that, at younger ages, German children who more consistently chose the optimal jar in the Bandit Jars task were also more perseverative on the responsive flexibility task. In line with our predictions, we observed a positive correlation between inhibition and Bandit Jars scores for BaYaka adolescents in the stable condition (*r_τ_* = −0.41, *p* = 0.04). Finally, we observed a positive correlation between Corsi and Bandit Jars performance in the stable condition for older Bandongo children (*r_τ_* = 0.54, *p* = 0.03) and adults (*r_τ_* = 36, *p* < 0.001).

### 4.3 Predictability, Variability, and Harshness Across Cultures

#### 4.3.1 Food Predictability

BaYaka adults had the highest likelihood of obtaining a predicted food item, this was statistically higher than German [Posterior predictive mean (95% CI) = 0.17 (0.07, 0.26)] and nearly Bandongo [0.08 (−0.01, 0.17)] participants. Interestingly, BaYaka adults were also more likely to predict a food item that was later not obtained, than both German [0.19 (0.11, 0.27)] and Bandongo [0.15 (0.07, 0.22)] participants. It should be noted that BaYaka predicted obtaining an average of 3.75 (SD = 1.01) unique items per day, while Bandongo and German participants predicted 2.42 (SD = 1.23) and 2.04 (SD = 1.43) items, respectively. Finally, both German [0.36 (0.25, 0.46)] and Bandongo [0.23 (0.13, 0.32)] participants were significantly more likely than BaYaka participants to obtain an item that was not planned. German participants were also significantly more likely than Bandongo participants to obtain an item that was not planned [0.13 (0.01, 0.24)]. Thus, Bandongo trended towards intermediate values between German and BaYaka participants. See Figures 4a and S6.

**FIGURE 4:**
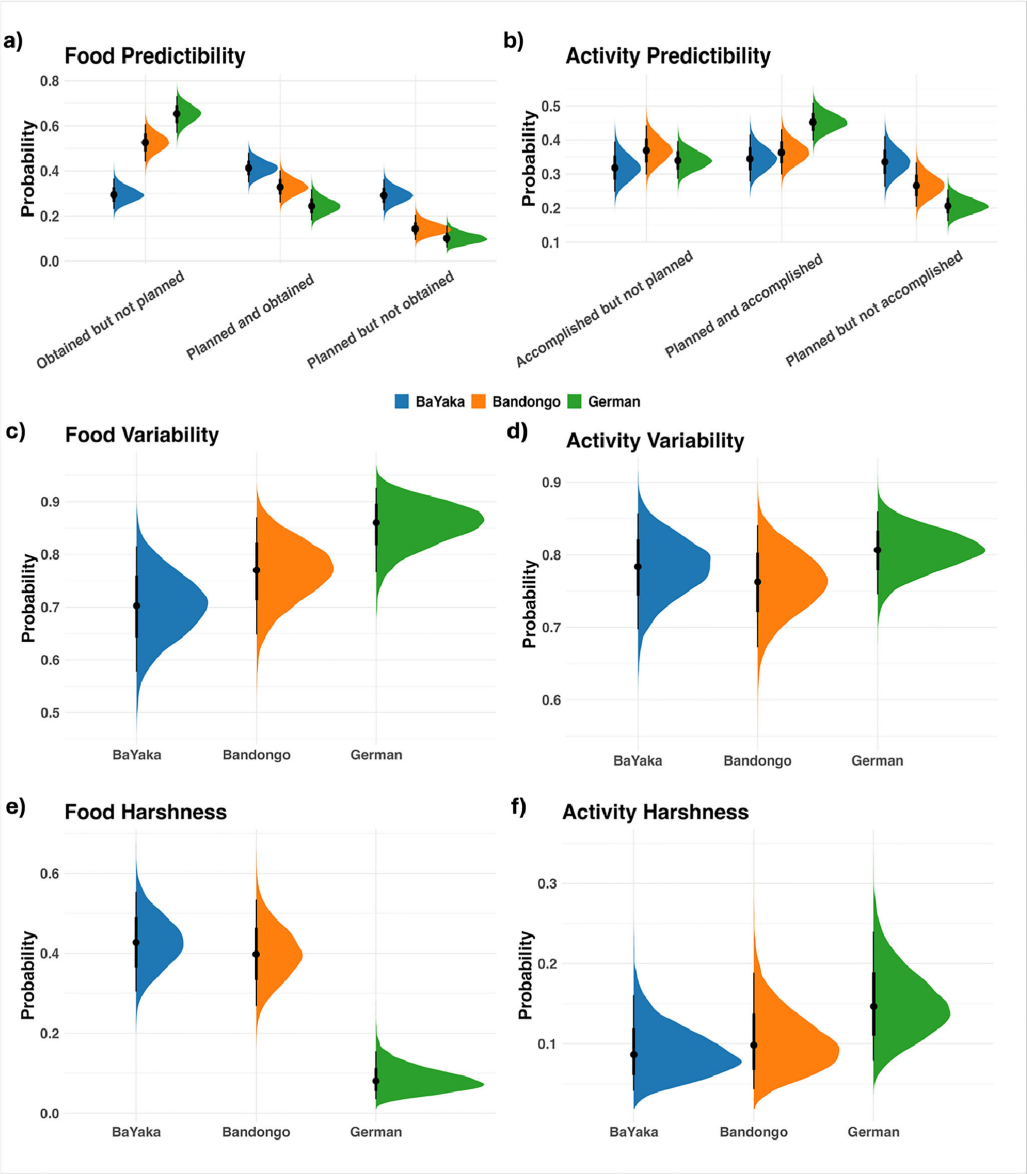
Exposure to predictability (a, b), variability (c, d), and harshness (e, f) in subsistence and daily activities across cultures.

#### 4.3.2 Activity Predictability

German participants were more likely to accomplish a planned activity than both BaYaka [Posterior predictive mean (95% CI) = 0.11 (0.02, 0.19)] and Bandongo [0.09 (0.00, 0.17)] participants. Bayaka participants were also significantly more likely to *not* accomplish a planned activity than German participants [0.13 (0.05, 0.22)]. There were no significant differences in participants’ likelihoods of accomplishing an activity that was not planned. BaYaka predicted accomplishing an average of 2.48 (1.11) unique activities per day, while Bandongo and German participants predicted 3.00 (sd = 1.28) and 4.00 (sd = 1.69) activities, respectively. See Figure 4b.

#### 4.3.3 Food Variability

Germans were significantly more likely to obtain a unique food item than BaYaka participants [Posterior predictive mean (95% CI) = 0.16 (0.03, 0.29)]. There were no differences between German and Bandongo participants, nor between Bandongo and BaYaka participants. See Figure 4c.

#### 4.3.4 Activity Variability

There were no significant cross‐cultural differences in participants’ likelihood of accomplishing a unique activity. See Figure 4d.

#### 4.3.5 Food Harshness

German participants were significantly less likely to report food harshness (e.g., either they or their family not having enough to eat) compared to both Bandongo [Posterior predictive mean (95% CI) = −0.32 (−0.17, −0.46] and BaYaka [−0.34 (−0.22, −0.47)] participants, who did not differ significantly from one another. See Figure 4e.

#### 4.3.6 Activity Harshness

There were no significant cross‐cultural differences in participants’ likelihood of reporting having dealt with a conflict or problem. See Figure 4f.

## 5 Discussion

This study aimed to assess the development of elective flexibility, and three related cognitive functions, across three cultures. We anticipated a U‐shaped trajectory in participants’ likelihood of switching jars in the Bandit Jars task under Variable conditions but found no age‐related or cultural differences in jar switching under Variable conditions. We did observe a U‐shaped trajectory in elective exploration in the Stable condition for those participants who used an RL approach: younger children were more explorative than older children and adolescents, but not adults. As predicted, we observed a decrease in switching with age under Stable conditions, across all cultures. We also found that children's elective flexibility performance (e.g., Bandit Jars scores) improved with age for German participants in both Stable and Variable conditions–and to some extent for Bandongo and BaYaka participants. Regarding participants’ everyday decision spaces, we found that Germans reported more unpredictability in food acquisition than both BaYaka and Bandongo participants, and more variability in the types of foods acquired than BaYaka participants. Additionally, Germans reported a low degree of food scarcity. Considering Germans’ higher scores and their strong tendency to use a reinforcement learning approach, this provides some real‐world support for the notion that greater exposure to unpredictable variability corresponds to increased elective flexibility, with more exposure to harshness corresponding with reduced elective flexibility. That said, we also observed a number of similarities across cultures in the development of elective flexibility, suggesting that aspects of this skillset develop fairly robustly across eco‐cultural environments.

One unanticipated finding was that German participants were far more likely to adopt a reinforcement‐learning approach in both the Stable and Variable conditions of the Bandit Jars task than participants from either Congolese culture, who instead relied heavily on random and repeat strategies. This finding both limits our ability to interpret cross‐cultural variation in Bandit Jars exploration and learning rates, and is itself a notable point of cultural variation. Interestingly, despite these differences in approach, there were minimal cross‐cultural differences in Bandit Jars score in the Variable (average difference = 0.10 units) compared to the Stable condition (average difference = 0.33 units). This is likely due to the Variable condition's inherent difficulty–even participants who used an RL approach seem to have barely performed above chance. Thus, one explanation for Congolese participants’ preference for non‐RL strategies is that the benefit of actively seeking out the best jars did not outweigh the effort, even in the Stable condition. In other words, the goal of finding the best jars may not have been motivating or relevant enough–which is entirely possible given that beads earned throughout the tasks did not possess inherent immediate value and were only later traded in for a small reward and the “goal” of the task (collecting enough water to fill a tube that resulted in receiving a bead, consecutively) was itself quite abstract. Indeed humans often rely on less cognitively demanding approaches when faced with difficult or obscure learning environments (Beilock and Decaro 2007; Frick et al. 2019; Mehlhorn et al. 2015; Mittelstädt et al. 2018; Schulz et al. 2018; Wu et al. 2022), and this aligns with the idea that satisficing (i.e., by using repeat and random approaches that are at least somewhat effective), rather than maximizing, serves as a valuable approach when facing high levels of uncertainty (Artinger et al. 2022). An interesting consideration is that Congolese participants’ greater exposure to food harshness, indicated by their interview responses, may contribute to more generally conservative problem‐solving approaches, as seen in two recent accounts of BaYaka and Bandongo children's cognitive flexibility (Vieira et al. 2025) and risk taking Vieira et al. (in press). By contrast, German participants reported obtaining more unpredictable food resources than BaYaka and Bandongo participants and more variable food resources than BaYaka participants, with a lower probability of not obtaining enough food compared to both Congolese groups. Although these interviews provided only a small window into participating communities’ exposures to predictability, variability, and harshness, these results are in line with the Constrained Flexibility Framework's assertion that elective flexibility may be suppressed by exposure to harshness (Pope‐Caldwell et al. 2024)—although research specifically testing this relationship is necessary.

Another likely and non‐mutually exclusive interpretation of the observed cross‐cultural differences in Bandit Jars approach is that, compared with Congolese participants, German participants were much more likely to have engaged in activities that, like the Bandit Jars task, feature abstract goals, opaque mechanisms (e.g., computerized), adult‐guided instructions, specific behavioral goals, and implicit rewards for maximizing or “being correct”. Given the known impact of contextual factors on cognitive performance across what are considered stable core capacities (Gaskins and Alcalá 2023; Miller et al. 2026; Munakata and Michaelson 2021), it is likely that cross‐cultural variation in participants' prior familiarity and comfort with these types of problem‐spaces dramatically shaped their Bandit Jars approach, possibly by allowing German participants to more readily detect structure in the underlying reward contingencies, especially in the Variable condition, thereby reducing their subjective uncertainty. Additionally, although all tasks required successful completion of a training phase, it is very likely that differing exposures to computerized and/or gamified experiments in general, across—and even within—cultures, contributed to a large portion of variability in participant performance on these tasks. Future decision‐making research aiming to minimize these confounds, by incorporating more culturally grounded games and activities guided by iterative piloting and participant feedback (see Gaskins and Alcalá 2023), or by explicitly including variation in exposure to formal education or similar testing scenarios, is crucial.

Critically, this cultural divergence in Bandit Jars approach occurred alongside a great deal of similarity in the developmental trajectory of elective flexibility. Notably, younger participants switched jars more frequently than older participants in the Stable condition in all three cultures and there were no cross‐cultural differences in jar switching in the Variable condition, at any age. Furthermore, although only significant for German participants, Bandit Jars scores improved with age in the Stable condition for all cultural groups—with estimated changepoints for the development of this skill occurring at nearly the same time for German and BaYaka participants (14.0 and 14.2 years, respectively), and, to some extent, Bandongo participants (∼10.2 years, though this estimate was relatively uncertain; Figure 3). Thus, despite substantial cross‐cultural differences in our participants’ schooling, subsistence, and characteristics of their everyday environments—the ability to find and selectively choose a stable, optimal strategy amongst several options improved similarly over the course of childhood, stabilizing around early adolescence. Considering how important this skill is for making good decisions in a complex environment, alongside our current findings, we suggest that elective flexibility under stable conditions, where the relative values of different strategies are fairly easy to discern and keep track of, develops early and relatively robustly across differing developmental environments.

We were unable to confidently analyze Congolese participants’ elective exploration and learning rates, due to their preference for non‐RL approaches. However, for German participants, we found that young children exhibited higher levels of exploratory behavior than older children and adolescents on the Bandit Jars task under Stable conditions and this likely corresponds to more random, as opposed to directed, exploration given younger children's overall lower scores and learning rates. This is in line with the theoretical assertion that early childhood is a period of enhanced exploration and strategy acquisition, where even unlikely hypotheses might be safely tested (Gopnik 2020). Our results mirror similar accounts of greater exploration and hypothesis testing in young children (Giron et al. 2023; Lucas et al. 2015; Sumner et al. 2019). Additionally, in line with Pope et al. (2015) and Ionescu (2017), we found that German adults’ elective exploration was not distinguishable from young children's, in the Stable condition. We anticipated this U‐shaped trajectory in exploratory behavior under Variable conditions; however, this was not observed, likely due to the continued need for exploration in the variable condition where underlying reward values changed frequently. A directed inquiry into how differing levels of variability impact explorative behavior over the course of development is warranted.

Our exploratory comparisons of the developmental changepoints and correlations between participants’ Bandit Jars and related cognitive functioning scores, found both similarities and differences across cultures and tasks. Across all cultures we found that inhibitory skill (as measured by the ECITT) improved until roughly age 10, suggesting a culturally robust developmental trajectory. We also found a positive correlation between Stable Bandit Jars score and inhibition for BaYaka adolescents, perhaps corresponding to the benefit of inhibiting jar‐switching within the stable condition. It is unclear why this was only significant for BaYaka participants within this age group; however, slight positive trends were observed for all other interpretable groups under 18‐years‐old. Additionally, we noted a positive correlation between Corsi and Bandit Jars performance in the stable condition for Bandongo older children and adults, suggesting that higher memory allowed for better maintenance of the optimal strategy; although this was not found for either German or BaYaka participants. German children's Bandit Jars performance under stable conditions and their CSST (responsive flexibility; only collected in Germany) performance had remarkably similar developmental trajectories. Both skillsets increased dramatically until approximately 14‐years‐of‐age. This points to similar underpinnings, which is not surprising given that, under stable conditions, elective flexibility simply requires identifying and selecting the best option–a fundamental component of task‐switching paradigms. Notably, younger German children exhibited a slightly negative correlation between Stable Bandit Jars scores and CSST performance. It is possible that prior to implementing an RL strategy, German children who were less perseverative (as measured by the CSST) may have also switched jars more frequently, further reducing their scores in the stable but not the variable condition. It is important to note that there was a great deal of uncertainty in Congolese participants’ Bandit Jars and cognitive functioning changepoint estimates, reflecting high inter‐individual differences. One important future direction will be to explore how local variation in exposure to formal education may account for these within‐group differences (Kroupin et al. 2025; Rawlings et al. 2023).

The current study took a multi‐methods approach, combining experiments and interviews to investigate how elective flexibility and related cognitive functions develop across three cultural contexts. We found similar developmental timing for improvements in elective flexibility across cultures under Stable conditions, alongside striking cross‐cultural differences in the use of reinforcement learning approaches in both conditions. Although developmental constraints undoubtedly shape cognitive trajectories, our findings highlight the equally important role of cultural experience in shaping decision‐making strategies, an interplay that remains underexamined (Amir and McAuliffe 2020; Nielsen and Haun 2016). Understanding how children learn to choose between strategies, and how these strategies grow into adult heuristics, is essential for developing a more comprehensive picture of human adaptive decision‐making. Future work should aim to disentangle how specific features of a child's environment, including uncertainty, harshness, and task familiarity, influence the development and deployment of flexible cognition.

## Author Contributions

**Sarah Pope‐Caldwell**: conceptualization, investigation, funding acquisition, writing – original draft, methodology, writing – review and editing, project administration, formal analysis, supervision, resources, visualization. **Elisa Felsche**: data curation, project administration, writing – review and editing, methodology. **Ardain Dzabatou**: methodology, project administration, resources, data curation. **Wilson Vieira**: methodology, writing – review and editing, data curation, supervision. **Luke Maurits**: formal analysis, writing – review and editing. **Dominik Deffner**: writing – review and editing, formal analysis. **Daniel Haun**: supervision, resources, funding acquisition, writing – review and editing.

## Funding

SPC was funded by the Leakey Foundation. SPC and DH were funded by the Max Planck Society for the Advancement of Science. DD was supported by the Deutsche Forschungsgemeinschaft(DFG, German Research Foundation) under Germany's Excellence Strategy—EXC 2002/1 “Science of Intelligence”—project number 390523135. The funders had no role in study design, data collection and analysis, decision to publish, or preparation of the manuscript.

## Conflicts of Interest

The authors have declared that no competing interests exist.

## Supporting information




**Supporting information**: desc70299‐sup‐0001‐SuppMat.docx

## Data Availability

The data, analysis scripts, and experiment files that support the findings of this study are openly available in https://osf.io/xj7av.

## Endnotes

1 Note: We did not have age information for 5 of the adult BaYaka men who participated in the interviews.

## References

[desc70299-bib-0001] Amir, D. , M. R. Jordan , K. McAuliffe , et al. 2020. “The Developmental Origins of Risk and Time Preferences Across Diverse Societies.” Journal of Experimental Psychology: General 149, no. 4: 4. 10.1037/xge0000675. 31512902

[desc70299-bib-0002] Amir, D. , and K. McAuliffe . 2020. “Cross‐Cultural, Developmental Psychology: Integrating Approaches and Key Insights.” Evolution and Human Behavior 41, no. 5: 430–444.

[desc70299-bib-0003] Artinger, F. M. , G. Gigerenzer , and P. Jacobs . 2022. “Satisficing: Integrating Two Traditions.” Journal of Economic Literature 60, no. 2: 598–635. 10.1257/jel.20201396.

[desc70299-bib-0004] Beilock, S. L. , and M. S. Decaro . 2007. “From Poor Performance to Success Under Stress: Working Memory, Strategy Selection, and Mathematical Problem Solving Under Pressure.” Journal of Experimental Psychology: Learning, Memory, and Cognition 33, no. 6: 6. 10.1037/0278-7393.33.6.983 17983308

[desc70299-bib-0005] Benoit, L. , S. Lew‐Levy , Y. R. Ouamba , and A. N. Crittenden . 2024. “Energetic Value Estimates of Wild and Domesticated Food Items Consumed by Congolese BaYaka Foragers.” Journal of Ecological Anthropology 24, no. 1: 1–12.

[desc70299-bib-0006] Berg, E. A. 1948. “A Simple Objective Technique for Measuring Flexibility in Thinking.” Journal of General Psychology 39, no. 1: 1. 10.1080/00221309.1948.9918159. 18889466

[desc70299-bib-0007] Blakey, E. , I. Visser , and D. J. Carroll . 2016. “Different Executive Functions Support Different Kinds of Cognitive Flexibility: Evidence From 2‐, 3‐, and 4‐Year‐Olds.” Child Development 87, no. 2: 2. 10.1111/cdev.12468. PMC4991299 26659697

[desc70299-bib-0008] Bonawitz, E. B. , T. J. van Schijndel , D. Friel , and L. Schulz . 2012. “Children Balance Theories and Evidence in Exploration, Explanation, and Learning.” Cognitive Psychology 64, no. 4: 4. 10.1016/j.cogpsych.2011.12.002 22365179

[desc70299-bib-0009] Boyette, A. H. , S. Lew‐Levy , and L. T. Gettler . 2018. “Dimensions of Fatherhood in a Congo Basin Village: A Multimethod Analysis of Intracultural Variation in Men's Parenting and Its Relevance for Child Health.” Current Anthropology 59, no. 6: 6. 10.1086/700717.

[desc70299-bib-0010] Bridgers, S. , Y. Wang , and D. Buchsbaum . 2019. “Children's Exploration as a Window Into Their Causal Learning.” Proceedings of the Annual Meeting of the Cognitive Science Society , 41, no. 0: 0.

[desc70299-bib-0011] Bruner, J. S. 1972. “Nature and Uses of Immaturity.” American Psychologist 27, no. 8: 687–708. 10.1037/h0033144.

[desc70299-bib-0012] Brydges, C. R. , A. M. Fox , C. L. Reid , and M. Anderson . 2014. “The Differentiation of Executive Functions in Middle and Late Childhood: A Longitudinal Latent‐Variable Analysis.” Intelligence 47: 34–43. 10.1016/j.intell.2014.08.010.

[desc70299-bib-0013] Bunge, S. A. , and J. D. Wallis . 2008. Neuroscience of Rule‐Guided Behavior. Oxford University Press.

[desc70299-bib-0500] Bürkner, P.‐C. 2017. “brms: An R Package for Bayesian Multilevel Models Using Stan.” Journal of Statistical Software 80, no. 1: 1.

[desc70299-bib-0014] Cheke, L. G. , E. Loissel , and N. S. Clayton . 2012. “How Do Children Solve Aesop's Fable?.” PLOS ONE 7, no. 7: 7. 10.1371/journal.pone.0040574. PMC3405099 22848384

[desc70299-bib-0506] City of Leipzig, Office for Statistics and Elections . 2023. https://static.leipzig.de/fileadmin/mediendatenbank/leipzig‐de/Stadt/02.1_Dez1_Allgemeine_Verwaltung/12_Statistik_und_Wahlen/Statistik/Leipzig_fb_Facts_and_Figures.pdf.

[desc70299-bib-0015] Cunningham, J. D. 1965. “Einstellung Rigidity in Children.” Journal of Experimental Child Psychology 2, no. 3: 3. 10.1016/0022-0965(65)90027-5.

[desc70299-bib-0016] Cusack, R. , M. Ranzato , and C. J. Charvet . 2024. “Helpless Infants Are Learning a Foundation Model.” Trends in Cognitive Sciences 28, no. 8: 8. 10.1016/j.tics.2024.05.001. PMC11310914 38839537

[desc70299-bib-0017] Cutting, N. , I. A. Apperly , J. Chappell , and S. R. Beck . 2014. “The Puzzling Difficulty of Tool Innovation: Why Can't Children Piece Their Knowledge Together?” Journal of Experimental Child Psychology 125: 110–117. 10.1016/j.jecp.2013.11.010. 24530037

[desc70299-bib-0018] Deffner, D. , V. Kleinow , and R. McElreath . 2020. “Dynamic Social Learning in Temporally and Spatially Variable Environments.” Royal Society Open Science 7, no. 12: 12. 10.1098/rsos.200734 PMC7813247 33489255

[desc70299-bib-0019] Diamond, A. 2013. “Executive Functions.” Annual Review of Psychology 64, no. 1: 1. 10.1146/annurev-psych-113011-143750. PMC4084861 23020641

[desc70299-bib-0501] Diekmann, Y. , D. Smith , P. Gerbault , et al. 2017. “Accurate Age Estimation in Small‐scale Societies.” Proceedings of the National Academy of Sciences 114, no. 31: 31. 10.1073/pnas.1619583114. PMC5547587 28696282

[desc70299-bib-0020] Doebel, S. , and P. D. Zelazo . 2015. “A Meta‐Analysis of the Dimensional Change Card Sort: Implications for Developmental Theories and the Measurement of Executive Function in Children.” Developmental Review 38: 241–268. 10.1016/j.dr.2015.09.001. PMC4778090 26955206

[desc70299-bib-0021] Forstmann, B. U. , M. Brass , I. Koch , and D. Y. V. Cramon . 2005. “Internally Generated and Directly Cued Task Sets: An Investigation With fMRI.” Neuropsychologia 43, no. 6: 943–952. 10.1016/j.neuropsychologia.2004.08.008. 15716164

[desc70299-bib-0022] Frick, A. , M. A. Brandimonte , and N. Chevalier . 2019. “Voluntary Task Switching in Children: Switching More Reduces the Cost of Task Selection.” Developmental Psychology 55, no. 8: 1615–1625. 10.1037/dev0000757. 31094559

[desc70299-bib-0023] Frick, A. , M. A. Brandimonte , and N. Chevalier . 2021. “Disentangling the Respective Contribution of Task Selection and Task Execution to Self‐Directed Cognitive Control Development.” Child Development 92, no. 4: 1309–1324. 10.1111/cdev.13479. 33351210

[desc70299-bib-0024] Frick, A. , and N. Chevalier . 2023. “A First Theoretical Model of Self‐Directed Cognitive Control Development.” Journal of Cognition and Development 24, no. 2: 191–204. 10.1080/15248372.2022.2160720.

[desc70299-bib-0025] Friedman, N. P. , and A. Miyake . 2017. “Unity and Diversity of Executive Functions: Individual Differences as a Window on Cognitive Structure.” Cortex 86: 186–204. 10.1016/j.cortex.2016.04.023. PMC5104682 27251123

[desc70299-bib-0026] Friedman, N. P. , A. Miyake , L. J. Altamirano , et al. 2016. “Stability and Change in Executive Function Abilities From Late Adolescence to Early Adulthood: A Longitudinal Twin Study.” Developmental Psychology 52, no. 2: 2. 10.1037/dev0000075. PMC4821683 26619323

[desc70299-bib-0027] Gaskins, S. , and L. Alcalá . 2023. “Studying Executive Function in Culturally Meaningful Ways.” Journal of Cognition and Development 24, no. 2: 260–279. 10.1080/15248372.2022.2160722.

[desc70299-bib-0028] German, T. P. , and M. A. Defeyter . 2000. “Immunity to Functional Fixedness in Young Children.” Psychonomic Bulletin & Review 7, no. 4: 4. 10.3758/bf03213010 11206213

[desc70299-bib-0029] Gettler, L. T. , D. Redhead , A. Dzabatou , and S. Lew‐Levy . 2023. “ bayaka Forager Food Sharing Networks in the Congo Basin: The Roles of Gender Homophily and Kin Sharing.” American Journal of Biological Anthropology 181, no. 1: 59–69. 10.1002/ajpa.24688.

[desc70299-bib-0030] Giron, A. P. , S. Ciranka , E. Schulz , et al. 2023. “Developmental Changes in Exploration Resemble Stochastic Optimization.” Nature Human Behaviour 7, no. 11: 1955–1967. 10.1038/s41562-023-01662-1. PMC10663152 37591981

[desc70299-bib-0032] Gopnik, A. 2020. “Childhood as a Solution to Explore–Exploit Tensions.” Philosophical Transactions of the Royal Society B: Biological Sciences 375, no. 1803: 1803. 10.1098/rstb.2019.0502 PMC7293160 32475327

[desc70299-bib-0033] Gopnik, A. , T. L. Griffiths , and C. G. Lucas . 2015. “When Younger Learners Can Be Better (or at Least More Open‐Minded) Than Older Ones.” Current Directions in Psychological Science 24, no. 2: 2.

[desc70299-bib-0034] Gweon, H. , and L. E. Schulz . 2008. “Stretching to Learn: Ambiguous Evidence and Variability in Preschoolersʼ Exploratory Play.” Proceedings of the 30th Annual Meeting of the Cognitive Science Society , 570–574.

[desc70299-bib-0035] Holmboe, K. , C. Larkman , C. de Klerk , et al. 2021. “The Early Childhood Inhibitory Touchscreen Task: A New Measure of Response Inhibition in Toddlerhood and Across the Lifespan.” PLoS One 16, no. 12: e0260695. 10.1371/journal.pone.0260695. PMC8638877 34855865

[desc70299-bib-0036] Hopper, L. M. , S. L. Jacobson , and L. H. Howard . 2020. “Problem Solving Flexibility Across Early Development.” Journal of Experimental Child Psychology 200: 104966. 10.1016/j.jecp.2020.104966. PMC7449664 32860967

[desc70299-bib-0037] Ionescu, T. 2017. “The Variability‐Stability‐Flexibility Pattern: A Possible Key to Understanding the Flexibility of the Human Mind.” Review of General Psychology 21, no. 2: 2.

[desc70299-bib-0038] Jacques, S. , and P. D. Zelazo . 2013. “On the Possible Roots of Cognitive flexibility.” In The Development of Social Cognition and Communication, 53–81. Psychology Press.

[desc70299-bib-0039] Kandza, V. H. , H. Jang , F. K. Ntamboudila , S. Lew‐Levy , and A. H. Boyette . 2023. “Intergroup Cooperation in Shotgun Hunting Among BaYaka Foragers and Yambe Farmers From the Republic of the Congo.” Human Nature 34, no. 2: 153–176. 10.1007/s12110-023-09448-0. PMC10354132 37099116

[desc70299-bib-0040] Kitanishi, K. 1995. “Seasonal Changes in the Subsistence Activities and Food Intake of the Aka Hunter‐Gatherers in Northeastern Congo.” African Study Monographs 16, no. 2: 73–118.

[desc70299-bib-0041] Kroupin, I. , H. E. Davis , E. Burdett , A. B. Cuata , V. Hartley , and J. Henrich . 2025. “The Cultural Construction of “Executive Function”.” Proceedings of the National Academy of Sciences of the United States of America 122, no. 27: e2407955122. 10.1073/pnas.2407955122. PMC12260431 40587792

[desc70299-bib-0042] Laureiro‐Martínez, D. , and S. Brusoni . 2018. “Cognitive Flexibility and Adaptive Decision‐Making: Evidence From a Laboratory Study of Expert Decision Makers.” Strategic Management Journal 39, no. 4: 4.

[desc70299-bib-0043] Legare, C. H. 2012. “Exploring Explanation: Explaining Inconsistent Evidence Informs Exploratory, Hypothesis‐Testing Behavior in Young Children.” Child Development 83, no. 1: 1. 10.1111/j.1467-8624.2011.01691.x. 22172010

[desc70299-bib-0044] Lew‐Levy, S. , and D. Amir . 2026. “Children as Agents of Cultural Adaptation.” Behavioral and Brain Sciences 49: e225. 10.1017/S0140525X24001377. 39721974

[desc70299-bib-0045] Lew‐Levy, S. , V. Kandza , H. Jang , et al. 2025. “BaYaka Forager and Bantu Fisher‐Farmer Adolescent Engagement With Intensifying Market Integration in the Republic of the Congo.” Journal of the Royal Anthropological Institute 31, no. 2: 118–143. 10.1111/1467-9655.70006.

[desc70299-bib-0046] Lew‐Levy, S. , L. Maurits , A. H. Boyette , et al. 2026. “The Social Learning and Development of Intra‐ and Inter‐Ethnic Sharing Norms in the Congo Basin.” PLOS ONE 21, no. 1: e0340388. 10.1371/journal.pone.0340388. PMC12851454 41604373

[desc70299-bib-0047] Lew‐Levy, S. , S. M. Pope , D. B. Haun , M. A. Kline , and T. Broesch . 2021. “Out of the Empirical Box: A Mixed‐Methods Study of Tool Innovation Among Congolese BaYaka Forager and Bondongo Fisher‐Farmer Children.” Journal of Experimental Child Psychology 211: 105223. 10.1016/j.jecp.2021.105223 34273734

[desc70299-bib-0048] Liquin, E. G. , F. Callaway , and T. Lombrozo . 2021. “Developmental Change in What Elicits Curiosity.” Proceedings of the Annual Meeting of the Cognitive Science Society 43, no. 43: 43.

[desc70299-bib-0049] Liquin, E. G. , and A. Gopnik . 2022. “Children Are More Exploratory and Learn More Than Adults in an Approach‐Avoid Task.” Cognition 218: 104940. 10.1016/j.cognition.2021.104940. 34715584

[desc70299-bib-0050] Lucas, C. G. , S. Bridgers , T. L. Griffiths , and A. Gopnik . 2015. “When Children Are Better (or at Least More Open‐Minded) Learners Than Adults: Developmental Differences in Learning the Forms of Causal Relationships.” Cognition 131, no. 2: 2. 10.1016/j.cognition.2013.12.010. 24566007

[desc70299-bib-0051] Luchins, A. S. 1942. “Mechanization of Problem Solving: The Effect of Einstellung.” Psychological Monographs 54, no. 6: 6.

[desc70299-bib-0052] Mata, R. , A. Wilke , and U. Czienskowski . 2013. “Foraging Across the Life Span: Is There a Reduction in Exploration With Aging?.” Frontiers in Neuroscience 7, no. 53: 1–7. 10.3389/fnins.2013.00053. PMC3627975 23616741

[desc70299-bib-0053] Maurer, A. , D. Guenther , and J. Kennedy . 2016. Automated Food Pellet Dispenser Model (Version 2)[3D Model] . NIH 3D. https://3d.nih.gov/entries/3728/2.

[desc70299-bib-0054] McElreath, R. 2020. Statistical Rethinking: A Bayesian Course With Examples in R and Stan *. Second Edition*. Chapman and Hall/CRC.

[desc70299-bib-0055] Mehlhorn, K. , B. R. Newell , P. M. Todd , et al. 2015. “Unpacking the Exploration–Exploitation Tradeoff: A Synthesis of Human and Animal Literatures.” Decision 2, no. 3: 3. 10.1037/dec0000033.

[desc70299-bib-0056] Miller, S. E. , L. Gago Galvagno , and Á. Elgier . 2026. “Understanding Contextual Variations in Executive Function Through Habits, Representations, and Reflection.” Frontiers in Developmental Psychology 4: 1711934. 10.3389/fdpys.2026.1711934.

[desc70299-bib-0057] Mittelstädt, V. , D. Dignath , M. Schmidt‐Ott , and A. Kiesel . 2018. “Exploring the Repetition Bias in Voluntary Task Switching.” Psychological Research 82, no. 1: 78–91. 10.1007/s00426-017-0911-5. 28871331

[desc70299-bib-0058] Miyake, A. , N. P. Friedman , M. J. Emerson , A. H. Witzki , A. Howerter , and T. D. Wager . 2000. “The Unity and Diversity of Executive Functions and Their Contributions to Complex “Frontal Lobe” Tasks: A Latent Variable Analysis.” Cognitive Psychology 41, no. 1: 1. 10.1006/cogp.1999.0734 10945922

[desc70299-bib-0059] Mullane, J. C. , and P. V. Corkum . 2007. “The Relationship Between Working Memory, Inhibition, and Performance on the Wisconsin Card Sorting Test in Children With and Without ADHD.” Journal of Psychoeducational Assessment 25, no. 3: 211–221. 10.1177/0734282906297627.

[desc70299-bib-0060] Munakata, Y. , and L. E. Michaelson . 2021. “Executive Functions in Social Context: Implications for Conceptualizing, Measuring, and Supporting Developmental Trajectories.” Annual Review of Developmental Psychology 3, no. 1: 139–163. 10.1146/annurev-devpsych-121318-085005. PMC11238700 38993653

[desc70299-bib-0061] Neldner, K. , J. Redshaw , S. Murphy , et al. 2019. “Creation Across Culture: Children's Tool Innovation Is Influenced by Cultural and Developmental Factors.” Developmental Psychology 55, no. 4: 4. 10.1037/dev0000672. 30640502

[desc70299-bib-0062] Nielsen, M. , and D. Haun . 2016. “Why Developmental Psychology Is Incomplete Without Comparative and Cross‐Cultural Perspectives.” Philosophical Transactions of the Royal Society B 371: 20150071. 10.1098/rstb.2015.0071 PMC4685517 26644590

[desc70299-bib-0063] Nweze, T. , and W. Nwani . 2020. “Contributions of Working Memory and Inhibition to Cognitive Flexibility in Nigerian Adolescents.” Developmental Neuropsychology 45, no. 3: 3. 10.1080/87565641.2020.1765169. PMC7116232 32412307

[desc70299-bib-0064] Pope, S. M. 2018. Differences in Cognitive Flexibility Within the Primate Lineage and Across Human Cultures: When Learned Strategies Block Better Alternatives [Doctoral dissertation]. Georgia State University & Aix Marseille University.

[desc70299-bib-0065] Pope, S. M. , A. Meguerditchian , W. D. Hopkins , and J. Fagot . 2015. “Baboons (Papio papio), but Not Humans, Break Cognitive Set in a Visuomotor Task.” Animal Cognition 18, no. 6: 6. 10.1007/s10071-015-0904-y PMC9836456 26194414

[desc70299-bib-0505] Pope‐Caldwell, S. 2019. Unpublished Survey [N = 96 participants].

[desc70299-bib-0066] Pope‐Caldwell, S. 2024. “How Dynamic Environments Shape Cognitive flexibility.” In The Evolution of Techniques: Rigidity and Flexibility in Use, Transmission, and Innovation, edited by M. Charbonneau , MIT Press.

[desc70299-bib-0067] Pope‐Caldwell, S. , D. Deffner , L. Maurits , T. Neumann , and D. Haun . 2024. “Variability and Harshness Shape Flexible Strategy‐Use in Support of the Constrained Flexibility Framework.” Scientific Reports 14, no. 1: 7236. 10.1038/s41598-024-57800-w. PMC10973413 38538731

[desc70299-bib-0068] Rawlings, B. S. , H. E. Davis , A. Anum , et al. 2023. “Quantifying Quality: The Impact of Measures of School Quality on Children's Academic Achievement Across Diverse Societies.” Developmental Science 27, no. 5: e13434. 10.1111/desc.13434. 37455378

[desc70299-bib-0069] Schulz, E. , C. M. Wu , Q. J. M. Huys , A. Krause , and M. Speekenbrink . 2018. “Generalization and Search in Risky Environments.” Cognitive Science 42, no. 8: 8. 10.1111/cogs.12695. 30390325

[desc70299-bib-0070] Smidts, D. P. , R. Jacobs , and V. Anderson . 2004. “The Object Classification Task for Children (OCTC): A Measure of Concept Generation and Mental Flexibility in Early Childhood.” Developmental Neuropsychology 26, no. 1: 385–401. 10.1207/s15326942dn2601_2. 15276901

[desc70299-bib-0071] Snyder, H. R. , and Y. Munakata . 2010. “Becoming Self‐Directed: Abstract Representations Support Endogenous Flexibility in Children.” Cognition 116, no. 2: 155–167. 10.1016/j.cognition.2010.04.007. PMC2900525 20472227

[desc70299-bib-0072] Sumner, E. , M. Steyvers , and B. W. Sarnecka . 2019. “It's Not the Treasure, It's the Hunt: Children Are More Explorative on an Explore/Exploit Task Than Adults.” In Proceedings of the Annual Meeting of the Cognitive Science Society 116, no. 41: 2891–2897.

[desc70299-bib-0073] Thompson‐Schill, S. L. , M. Ramscar , and E. G. Chrysikou . 2009. “Cognition Without Control: When a Little Frontal Lobe Goes a Long Way.” Current Directions in Psychological Science 18, no. 5: 5. 10.1111/j.1467-8721.2009.01648.x. PMC2855545 20401341

[desc70299-bib-0074] Ueltzhöffer, K. , D. J. N. Armbruster‐Genç , and C. J. Fiebach . 2015. “Stochastic Dynamics Underlying Cognitive Stability and Flexibility.” PLOS Computational Biology 11, no. 6: 6. 10.1371/journal.pcbi.1004331 PMC4466596 26068119

[desc70299-bib-0075] Veen, J. , H. Jang , D. Raubenheimer , et al. 2023. “Development of Embodied Capital: Diet Composition, Foraging Skills, and Botanical Knowledge of Forager Children in the Congo Basin.” Frontiers in Ecology and Evolution 11: 935987. 10.3389/fevo.2023.935987.

[desc70299-bib-0076] Vieira, W. , L. Maurits , A. Dzabatou , D. B. M. Haun , and S. Pope‐Caldwell . in press. “Development of Explorative Search and Risk Tolerance in BaYaka, Bandongo, and German Children.” Developmental Psychology .

[desc70299-bib-0077] Vieira, W. , S. Pope‐Caldwell , A. Dzabatou , L. Maurits , M. Padberg , and D. Haun . 2025. “Social Learning Leads to Inflexible Strategy Use in Children Across Three Societies.” Scientific Reports 15, no. 1: 30281. 10.1038/s41598-025-15400-2. PMC12361463 40826217

[desc70299-bib-0078] Watanabe, S. 2010. “Asymptotic Equivalence of Bayes Cross Validation and Widely Applicable Information Criterion in Singular Learning Theory.” Journal of Machine Learning Research 11: 3571–3594.

[desc70299-bib-0079] Wu, C. M. , E. Schulz , T. J. Pleskac , and M. Speekenbrink . 2022. “Time Pressure Changes How People Explore and Respond to Uncertainty.” Scientific Reports 12, no. 1: 1. 10.1038/s41598-022-07901-1. PMC8904509 35260717

[desc70299-bib-0080] Xu, J. , L. Wood , C. Sutherland , et al. 2026. “Parental Perspectives on Children's Social Learning in Four Cultures.” Frontiers in Psychology 17: 1845262. 10.3389/fpsyg.2026.1845262. PMC13294349 42369302

[desc70299-bib-0081] Zelazo, P. D. 2006. “The Dimensional Change Card Sort (DCCS): a Method of Assessing Executive Function in Children.” Nature Protocols 1, no. 1: 1. 10.1038/nprot.2006.46. 17406248

[desc70299-bib-0082] Zelazo, P. D. , D. Frye , and T. Rapus . 1996. “An Age‐related Dissociation Between Knowing Rules and Using Them.” Cognitive Development 11, no. 1: 1. 10.1016/s0885-2014(96)90027-1.

