# Supplementary material for: Development of Elective Flexibility and Related Cognitive Functions Across Three Cultures
Source: Dev Sci. 2026 Nov 3;30(1):e70299. doi: 10.1111/desc.70299 (PMC13731931; doi:10.1111/desc.70299)
Supplement: Supplementary file 1 [file DESC-30-e70299-s001.docx]

# **Supplementary Figures**

**Supplementary Figure 1**. Age distribution of participants in the task battery by culture.

**Supplementary Figure 2.** a) The bimodal distribution of θ RL scores leading to the decision to use θ RL > 0.8 as the cutoff point for θ values we considered indicative of using an RL strategy. b) Posterior halfeye plot showing the likelihood of θ RL > 0.8 across cultures for each age group.

**Supplementary Figure 3.** a) Example track showing the number of unique 10m grids intersected by a Bandongo participant, b) example track showing the number of unique 10m grids intersected by a BaYaka participant, c) halfeye plot for the proportion of unique cells, per individual, visited across GPS tracks.

**Supplementary Figure 4.** Correlation matrices between age and the related cognitive functioning scores and Bandit outcome measures, across cultures.

**Supplementary Figure 5.** Kendall’s rank correlation matrices between Bandit Jars score and memory (Corsi), inhibition (ECITT), and switching (CSST) cognitive functions, across cultures and age groups. Note, we did not interpret correlations from groups containing fewer than 10 participants.

**Supplementary Figure 6.** The raw data from participant interviews grouped into categories and coded for whether or not it was planned, obtained, or both. *Note: We have chosen not to publish the specific items interviewees reported, in an abundance of caution for their privacy; however, these data can be made available in some capacity upon request.*

# **Supplementary Methods**

## **Cognitive Functioning Scores**

### ***ECITT: Inhibition***

Participants’ inhibition scores were calculated as the difference in Linear Integrated Speed and Accuracy Scores (LISAS; see Vandierendonck, 2017), which combine response times and error rates, between prepotent (75% of trials, no need to inhibit the prepotent response) and inhibitory ECITT trials (25% of trials, prepotent response is incorrect, requires inhibition). In a slight deviation from our pre-registration, we filtered out the first trial response times (median = 1634ms; SD = 2136.8), which were frequently longer than the median (1052ms; SD = 1156.9) and all trials where response times were greater than 8 seconds ( N_Trials_ = 61, Median = 9881ms; SD = 5162.467; Max = 36651). This was done prior to analysis.

### ***CSST: Switching Score***

Participants’ switching scores,–the deficit in accuracy and response times associated with switching strategies– were calculated as the difference in LISAS between trials in which the previous rule was repeated versus switched during the Mixed block of the Color-Shape Task. While piloting the CSST with BaYaka and Bandongo participants, we found that pilot participants did not consistently pass the CSST training levels. This is likely due to the abstract nature of the rule presentation (a graphic banner along the top of the screen) which has no ecological or cultural significance in that context. Thus, we opted not to collect CSST data with either BaYaka or Bandongo communities.

### ***Corsi: Spatial Memory Score***

Participants’ spatial memory scores were calculated as the maximum number of levels (1-9) successfully completed by a participant, at least once.

## **Deviations from Pre-Registration**

### ***No CSST data collected in Congo***

During initial data collection for the CSST in Congo, we noticed that a number of our research assistants required substantial assistance to complete the CSST task. Given that our team is well-versed in cognitive tasks like these, this was somewhat surprising. When asked, they described the abstract cue (the banner at the top of the screen that is either alternating gray shapes or reference colors) indicating which rule to sort by as being very difficult to understand. We attempted to find alternative instructions/translations, as well as an alternative visual cue. However, after substantial effort and piloting with 7 participants [2 Bandongo (N_female_=1, ages 7.98-11.4 years) and 5 BaYaka (N_female_=0, ages 8.49-17.5 years)], of which only two passed the comprehension checks, we opted to drop this task from the battery.

### ***No inclusion of cognitive functioning scores in the primary models***

We had originally planned to run models structured as:

*outcome variable ~ 1 + sex + age*condition + age*CSST_switching + age*ECITT + age*mo(Corsi) + (1 + condition | subject) + (1 + sex + age*condition + age*CSST_switching + age*ECITT + age*mo(Corsi) | culture)*

However, we later realized that inclusion of the cognitive functioning scores could lead to a potential confound, where variance attributable to cognitive functioning might mediate, or collinearly absorb, age-related variance in elective flexibility. See Supplementary Figures 4 and 5.

Additionally, this would have limited our models to only those participants who completed completed all tasks–Bandit Jars, ECITT, CSST (for German participants) and Corsi–which is 83 Bandongo (N_Female_= 36, 6.5-71.0 years-old), 48 BaYaka (N_Female_ = 22, 4.5-62.1 years-old), and 163 Germans (N_Female_= 83, 4.2-81 years-old). Note, this would have excluded [8 Bandongo (4.0-69.0 years-old), 18 BaYaka (3.5-57.5 years-old) and 5 Germans (4.0-11.9 years-old)].

Instead, we ran exploratory “changepoint” analyses and Kendall’s rank correlation analyses to compare and contrast developmental trajectories between elective flexibility and the related cognitive functioning scores.

***GPS tracking***

In the Republic of the Congo, GPS tracking was done for all interviewed participants using Garmin Instinct Solar watches, set to *Hike* mode (the closest matching activity option) using GPS + GLONASS satellite systems. Watch faces were covered using a plastic token glued into a silicon watch protector and buttons were placed in lock-mode prior to distribution. All participants were informed of the GPS functioning prior to their first day of data collection; specifically, they were told that it could “see where they went, but not what they were doing” and that they could opt out at any time, for any reason. We answered any remaining questions or concerns and asked if they would still like to participate in that part of the study.

In Germany, we were unable to collect GPS tracks due to substantial building interference found during piloting. In total we recorded 102 GPS tracks from the 43 interview participants, between September 9th 2021 - October 10th 2021. Of these recordings, 10 were corrupt and unable to be included in analyses, leaving 92 tracks [Mean (SD) number of days per participant = 2.04 (0.79)_BaYaka_ ; 2.28 (0.75)_BaYaka_ ] for analysis.

To measure variability in participants’ land use, we identified participants for which we had data on more than one day (N_1 Day_ = 10, N_2 Days_ = 16, N_3 Days_ = 17) and calculated the number of 10m x 10m cells that each participant passed through during only one day, as a proportion of the total number of 10m x 10m cells that they intersected across all recorded days using the *xtracks* package in R (Wood, 2022). We then ran a series of regressions to predict the proportion of unique cells that were single-day visits. Models 13.1 included sex and the number of GPS tracks (days) for that participant. Models 13.2 also included culture as a predictor.

### Location Variability

There were no significant cross-cultural differences in the number of 10m cells intersected by Congolese participants’ GPS tracks on only one day, as a proportion of the total number of unique cells intersected. See Supplementary Figure 3.
